# The Detection of *Candida* Species in Patients with Halitosis

**DOI:** 10.1155/2014/857647

**Published:** 2014-08-24

**Authors:** Chihiro Koga, Masahiro Yoneda, Keisuke Nakayama, Satoru Yokoue, Mariko Haraga, Tomoko Oie, Arisa Suga, Fumiko Okada, Hiroshi Matsuura, Fumitake Tsue, Nao Suzuki, Takao Hirofuji

**Affiliations:** ^1^Department of Oral and Maxillofacial Surgery, Center for Oral Diseases, Fukuoka Dental College, 3-2-1 Hakataekimae, Hakata-ku, Fukuoka 812-0011, Japan; ^2^Section of General Dentistry, Department of General Dentistry, Fukuoka Dental College, 2-15-1 Tamura, Sawara-ku, Fukuoka 814-0193, Japan

## Abstract

To examine the effects of* Candida* on halitosis, the carrier state of* Candida* was examined in patients who made a visit with a chief complaint of halitosis.* Methods*. Subjects were 123 patients (42 males and 81 females) who visited our clinic, with a chief complaint of halitosis. Their average age was 45.8 years. To examine halitosis, an organoleptic test was conducted, and volatile sulfur compounds (VSCs) were measured by gas chromatography. Tongue-coating samples collected at the initial visit were cultured in CHROMagar* Candida* medium. The results of a* Candida* culture test, an organoleptic test, and VSC measurements were examined.* Results*. The male-to-female ratio of the patients was about 1 : 2. Patients with severe halitosis accounted for less than 20%. In the* Candida* culture test, the positive rate was about 25.2%, and* C. albicans* was the most frequently detected. Two kinds of* Candida* species were detected in 75% (6/8) of the strongly* Candida*-positive group. The VSC measurements were correlated with the* Candida* culture test results. Methyl mercaptan concentration was higher in the strongly* C. albicans*-positive group or the subjects having two kinds of* Candida* species.* Conclusion*. We suggest that imbalance of oral microbial community exists in the strongly *Candida*-positive group.

## 1. Introduction

The agents of halitosis are volatile sulfur compounds (VSCs), which are mainly composed of hydrogen sulfide and methyl mercaptan [1–4]. Bacteria associated with periodontal diseases have a strong ability to produce VSCs. In fact, halitosis is often improved by treating periodontal diseases [5–7]. However, some cases of halitosis are not improved by treating periodontal diseases, suggesting other causes. A coated (furred) tongue has recently been considered as a cause of halitosis [8]. Various microorganisms exist on a coated tongue. Of these,* Candida* is the most frequently detected, reflecting the oral environment [9, 10].* Candida* may be involved in halitosis. To examine the effects of* Candida* on halitosis, the carrier state of* Candida* was examined in patients who made a visit with a chief complaint of halitosis.

## 2. Materials and Methods

Subjects were 123 patients (42 males and 81 females) who visited the Center for Oral Diseases, Fukuoka Dental College, with a chief complaint of halitosis between April 2012 and June 2013 and from whom complete data and consent were obtained for publication. Their average age was 45.8 years (male: 45.2; female: 46.1). An organoleptic test was conducted, and VSCs were measured by gas chromatography.

Tongue-coating samples collected from behind the center of the dorsal surface of the tongue at the initial visit were cultured in CHROMagar* Candida* agar medium. The results of a* Candida* culture test, an organoleptic test of halitosis, and VSC measurements by gas chromatography were examined.

Permission for this study was obtained from the Ethics Committee for Clinical Research of Fukuoka Dental College and Fukuoka College of Health Sciences (approval number 125).

### 2.1. Malodor Assessment

For each patient, malodor was assessed and a clinical examination was performed at the same time of day at least 5 hours after eating, drinking, chewing, smoking, and brushing or rinsing the mouth. The severity of oral malodor in each individual was determined using an organoleptic test (OLT) and gas chromatography (model GC14B; Shimadzu Works, Kyoto, Japan). For the OLT, each patient was instructed to exhale through the mouth with moderate force into a Teflon sampling bag (GL Science, Tokyo, Japan) for 2 to 3 seconds to prevent the dilution of mouth odor with lung and room air. This procedure was repeated until approximately 1 L of breath sample was obtained. Two of the 3 evaluators (with training and experience in calibration tests) then estimated the odor at a distance of 10 cm from the sampling bag. The OLT scores were estimated on a scale of 0 to 5 (0, no odor; 1, questionable odor; 2, slight malodor; 3, moderate malodor; 4, strong malodor; 5, severe malodor) [11], and mean scores given by the different judges were used. The percentage agreement in the OLT scores among the 3 evaluators always exceeded 75.0% (*K* = 0.50). The threshold level for genuine halitosis was defined as an OLT score of 2 or higher according to the experimental criteria [11]. For the gas chromatographic measurements, the subjects were asked to remain quiet with a closed mouth for 30 seconds, after which mouth air (10 mL) was aspirated using a gas-tight syringe. These samples were injected into a gas chromatograph column at 70°C. A glass column was packed with 25% *β*, *β*9-oxydipropionitrile on a 60- to 80-mesh Chromosorb W A W-DMCS-ST device (Shimadzu, Kyoto, Japan) fitted with a flame photometric detector. The concentration of each sulfur compound was determined based on the values for standard H_2_S, CH_3_SH, and CH_3_SCH_3_ gas prepared with a PD-1B permeameter (GL Science, Tokyo, Japan). The level of total VSCs was defined as the total concentrations of H_2_S, CH_3_SH, and CH_3_SCH_3_. The threshold levels for genuine halitosis were defined as 2.5 ng or more per 10 mL of total VSCs in mouth air by gas chromatography, according to previous reports [12, 13].

### 2.2. Isolation of* Candida*

For the* Candida* culture test, tongue-coating samples were collected from behind the center of the dorsal surface of the tongue. Isolation of* Candida* from the samples was performed with BBLTM CHROMagarTM* Candida* medium (Becton Dickinson, Sparks, MD, USA) [14, 15]. This medium was selected since each colony develops a specific colorization that renders it readily identifiable. The samples were then used to inoculate the medium and aerobically cultured for 3 days at 38°C. Four* Candida* species (*C. albicans*,* C. tropicalis*,* C. krusei*, and* C. glabrata*) were identified based on the colors of the cultured colonies.

### 2.3. Presence or Absence of* Candida*

The presence of* Candida* was determined by counting the number of colonies and then classifying them into 3 levels as follows: no detection (ND), (0); positive, (1–100); and strongly positive, (≥101) [16].

### 2.4. Statistical Processing

Statistical processing was conducted with *χ* ^2^ and unpaired *t*-tests.

## 3. Results

**Figure 1:**
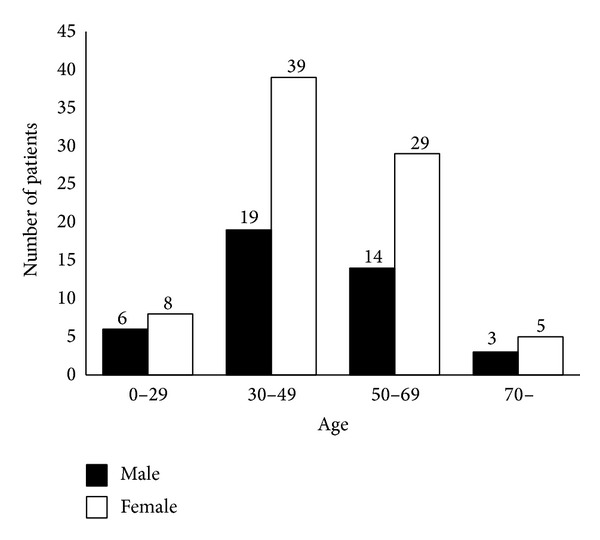
The sex and age distributions of the patients. Total 123 patients (42 males and 81 females).

Patient backgrounds and organoleptic test results are as follows. The sex and age distributions of the patients demonstrated that women were two times more common than men and that those in their 30s and 60s were more common (Figure 1).

The organoleptic test results demonstrated that mild halitosis (OLT scores: 1 and 2) was more common in women than in men, with a significant difference between men and women when classified into OLT scores 1, 2, and 3–5 (Table 1). The percentages of patients with severe halitosis (OLT scores: 4 and 5) were 16.7% for men and 18.5% for women, showing no significant difference. In total, patients with severe halitosis accounted for less than 20%.

*Candida* culture results are as follows. Of the 123 patients, 31 were positive (positive rate: about 25.2%;* C. albicans*: 17 cases,* C. glabrata*: 4 cases,* C. tropicalis*: 3 cases,* C. krusei*: 1 case,* C. albicans* and* tropicalis*: 4 cases, and* C. albicans* and* glabrata* or* krusei*: 1 case). Of the 31 positive cases, 23 cases were positive (1–100 colonies), while 8 cases were strongly positive (101 or more colonies). Two kinds of* Candida *species were detected in 75% (6/8) of the strongly* Candida*-positive group.* C. albicans *was combined in all the groups from which two kinds of* Candida *species were detected, and it was more predominant than another* Candida *species (Table 2).

**Figure 2:**
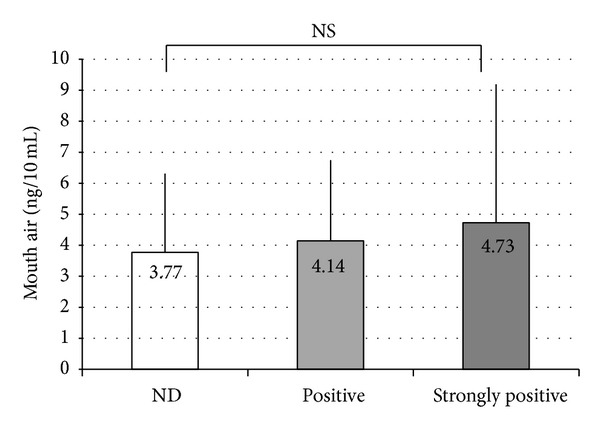
Correlations between the total VSCs measurements and* Candida* culture test results. NS: not significant. Vertical line: SD. Statistical comparison was performed using unpaired *t*-tests.

**Figure 3:**
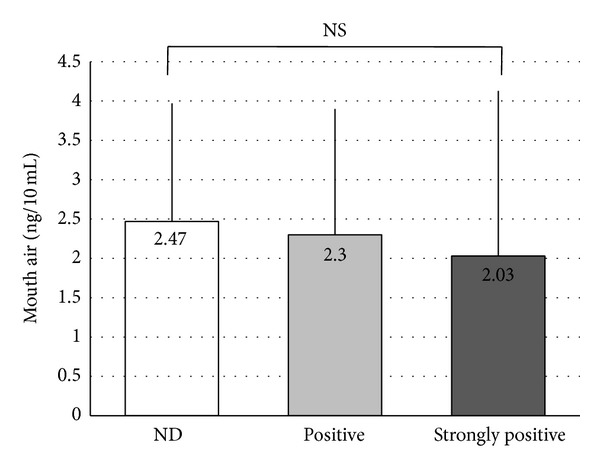
Correlations between the hydrogen sulfide measurements and* Candida* culture test results. NS: not significant. Vertical line: SD. Statistical comparison was performed using unpaired *t*-tests.

**Figure 4:**
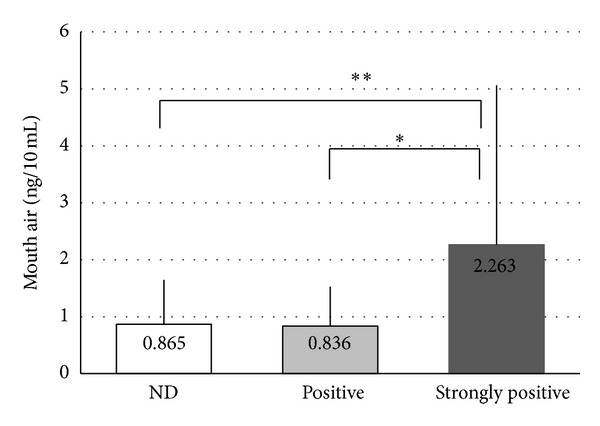
Correlations between the methyl mercaptan measurements and* Candida* culture test results. *P < 0.05, **P < 0.01. Vertical line: SD. Statistical comparison was performed using unpaired *t*-tests.

**Figure 5:**
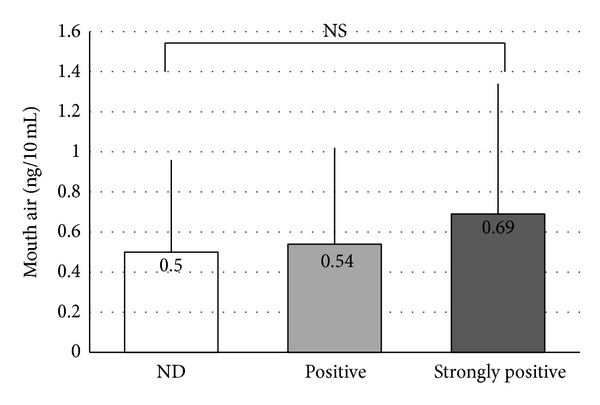
Correlations between the dimethyl sulfide measurements and* Candida* culture test results. NS: not significant. Vertical line: SD. Statistical comparison was performed using unpaired *t*-tests.

Correlations between the VSC measurements and* Candida* culture test results were as follows. The culture results were divided into three stages (ND, positive, and strongly positive) to examine correlations with results for total VSCs (Figure 2), hydrogen sulfide (Figure 3), dimethyl sulfide (Figure 4), and methyl mercaptan (Figure 5). No significant difference was observed in total VSCs, hydrogen sulfide, and dimethyl sulfide. Methyl mercaptan showed a significant increase in the strongly positive group, as compared with the other groups.

## 4. Discussion

The male-to-female ratio of the patients who made a visit was about 1 : 2, suggesting that women were more concerned about halitosis than men. This is as previously reported [17]. The organoleptic test results demonstrated that no or mild halitosis (OLT scores: 1 and 2, resp.) was more common among women, suggesting that women are more concerned about halitosis and undergo a halitosis test if they are concerned about even mild halitosis. The percentages of severe halitosis were less than 20% for both men and women. Thus, those without sever halitosis made a visit, suggesting a marked concern about halitosis. The previous study reported that 27.3% of the subjects who complained of halitosis were classified in the neurotic mental status by a psychological test and that the difficulties experienced by the subjects with neurotic tendencies and no oral malodor may manifest primarily in psychosomatic symptoms [18].

Of* Candida *species,* C. albicans *was the most common. This is as previously reported [16]. The detection rate of* Candida *was 25%, lower than previous results (about 40%) [19]. The patients who received a halitosis test seemed to be more careful about daily oral hygiene. A larger amount of methyl mercaptan was produced in the strongly positive group. Neither hydrogen sulfide nor dimethyl sulfide was correlated with the amount of* Candida*. Methyl mercaptan is formed by some members of the genera* Fusobacterium*,* Bacteroides*,* Porphyromonas,* and* Eubacterium*, which are recognized as periodontopathic bacteria [20]. Although methyl mercaptan was correlated with the increase of* Candida *in the present study, whether or not* Candida *directly generates methyl mercaptan is unknown. Under symbiosis with* Candida*, bacterial species associated with the production of methyl mercaptan may grow, and in old previous report,* Candida *species have an ability to produce VSCs [21].

Ninety percent of causes of halitosis are associated with oral components [2]. Tongue coating is understood to be a primarily important site from which VSCs are generated. The previous study found that the periodontal patients had greater tongue coating than healthy controls [6]. Proportions of some periodontopathic bacteria in tongue coating were correlated strongly with the concentrations of hydrogen sulfide and methyl mercaptan [22]. A tongue coating is formed with food and bacteria, facilitating the growth of* Candida*.* Candida *species are normal inhabitants in the oral cavity, and therefore the only presence of* Candida *species may not affect the production of VSCs. In fact, there were no differences of VSC levels between the* Candida*-negative group and the* Candida*-positive group. On the other hand, the overgrowth of* Candida *in the oral cavity is caused by oral environmental deterioration (e.g., immune compromise due to AIDS, aging, cancer, decreased salivation, periodontal diseases, steroid use, and antibiotic use) [19, 23]. A disturbance in the balance between commensal microbiota and the host immune system results in a switch from a healthy state to a diseased state even in the limited oral niche. Methyl mercaptan concentration was higher in the strongly* C. albicans*-positive group or the subjects having two kinds of* Candida *species, suggesting that imbalance of oral microbial community. In the future, the role of* Candida *species on oral malodor will be more revealed by the examinations including the amount of tongue coating, bacterial species in the tongue coating, periodontal conditions, and bacterial species in periodontal pockets.

## Figures and Tables

**Table 1:** The organoleptic test (OLT) results.

	OLT scores							
	1	2	3	4	5	Total	1, 2/1–5	
Males	5	9	21	7	0	42	14/42	∗
Females	21	21	24	12	3	81	42/81	

Total	26	30	45	19	3	123	56/123	

*P < 0.05: significant difference between men and women when classified into OLT scores 1, 2, and 3–5. Statistical comparison was performed using a chi-square test.

**Table 2:** *Candida* culture test results.

*Candida* species	Number of patients (strongly positive∗)
ND∗∗	92
*C. albicans *	17 (2)
*C. albicans* + *C. glabrata *	4 (4)
*C. albicans* + *C. tropicalis *	1 (1)
*C. albicans* + *C. krusei *	1 (1)
*C. glabrata *	4
*C. tropicalis *	3
*C. krusei *	1

Total	123

∗The patient whose total colony count was 101 or more.

∗∗ND: no detection.
